# A species independent universal bio-detection microarray for pathogen forensics and phylogenetic classification of unknown microorganisms

**DOI:** 10.1186/1471-2180-11-132

**Published:** 2011-06-14

**Authors:** Shamira J Shallom, Jenni N Weeks, Cristi L Galindo, Lauren McIver, Zhaohui Sun, John McCormick, L Garry Adams, Harold R Garner

**Affiliations:** 1 Virginia Bioinformatics Institute, Virginia Tech, Blacksburg, VA, USA; 2 St. Jude Children's Research Hospital, 262 Danny Thomas Place, Memphis, TN, USA; 3 Department of Veterinary Pathobiology, College of Veterinary Medicine, Texas A & M University, College Station, TX, USA

## Abstract

**Background:**

The ability to differentiate a bioterrorist attack or an accidental release of a research pathogen from a naturally occurring pandemic or disease event is crucial to the safety and security of this nation by enabling an appropriate and rapid response. It is critical in samples from an infected patient, the environment, or a laboratory to quickly and accurately identify the precise pathogen including natural or engineered variants and to classify new pathogens in relation to those that are known. Current approaches for pathogen detection rely on prior genomic sequence information. Given the enormous spectrum of genetic possibilities, a field deployable, robust technology, such as a universal (any species) microarray has near-term potential to address these needs.

**Results:**

A new and comprehensive sequence-independent array (Universal Bio-Signature Detection Array) was designed with approximately 373,000 probes. The main feature of this array is that the probes are computationally derived and sequence independent. There is one probe for each possible 9-mer sequence, thus 4^9 ^(262,144) probes. Each genome hybridized on this array has a unique pattern of signal intensities corresponding to each of these probes. These signal intensities were used to generate an un-biased cluster analysis of signal intensity hybridization patterns that can easily distinguish species into accepted and known phylogenomic relationships. Within limits, the array is highly sensitive and is able to detect synthetically mixed pathogens. Examples of unique hybridization signal intensity patterns are presented for different *Brucella *species as well as relevant host species and other pathogens. These results demonstrate the utility of the UBDA array as a diagnostic tool in pathogen forensics.

**Conclusions:**

This pathogen detection system is fast, accurate and can be applied to any species. Hybridization patterns are unique to a specific genome and these can be used to decipher the identity of a mixed pathogen sample and can separate hosts and pathogens into their respective phylogenomic relationships. This technology can also differentiate between different species and classify genomes into their known clades. The development of this technology will result in the creation of an integrated biomarker-specific bio-signature, multiple select agent specific detection system.

## Background

Rapid, accurate and sensitive detection of bio-threat agents requires a broad-spectrum assay capable of discriminating between closely related microbial or viral pathogens. In cases where a biological agent release has been identified, forensic analysis demands detailed genetic signature data for accurate strain identification and attribution. Identification of genetic signatures for detection coupled with identification of pathogenic phenotypes would provide a robust means of discriminating pathogens from closely related but benign species [1].

Current forensics methods based on bacteriological, serological, biochemical and genomic strategies have been used to detect pathogens using serological methods [2], PCR [3], real time PCR [4,5] and Multi-loci VNTR (variable-number tandem repeats) or MLVA [6-9]. Although bacteriological culture of *Brucella *spp. from blood, milk, fetal fluids and tissues, or other host tissues remain the 'gold standard' for diagnosis, bacteriologic culture has reduced sensitivity, is labour intensive, time consuming, typically requiring two weeks, and is a risk for laboratory personnel [5]. Serological assays, such as Rose Bengal, a rapid plate agglutination diagnostic test, is currently used for diagnosing infection with *Brucella *species in the field [2], however serological tests frequently have reduced specificity due to cross reactivity with other bacteria. Specific antibodies are required to be present at sufficiently high level and may require several weeks to develop before they are detectable. PCR based methods are used for epidemiological trace back and strain specific identification [3]. Although rapid in nature, specific primers are required for specific genes from these genomes or 16S rRNA genes or VNTR (variable-number tandem repeats) in a given genome. Real time PCR based methods have been used to identify *Brucella *species using IS711, bcsp31 and per target genes [4,5]. In addition, assays based on single-nucleotide polymorphisms have been developed for identification of *Brucella *isolates at the species level. These SNPs have been used to classify isolates into known *Brucella *species [10]. Recently MLVA or multi-loci VNTR (Variable-number tandem repeats) a genotype-based typing method and has been used as an epidemiological classification and SNP identification method for *Brucella *isolates in a field population [6-9]. MLVA method is used to understand the genetic diversity in polymorphic loci and to establish taxonomic relationships between different biovars of *Brucella*. It is used for microbial typing and epidemiologic studies by amplifying loci which are specific to a given genome and sequencing these regions. This is a powerful approach and is being used to create phylogenetic relationships and discovery of single nucleotide polymorphisms in independent loci from different *Brucella *isolates [7].

Array based approaches for forensic detection utilizes genome specific ribosomal RNA genes, genome specific PCR markers or oligonucleotide probes. Arrays from rRNA are derived from a combination of rRNA genes from a given set of organisms of high priority. Universal PCR is used to amplify one or more universal genes, including 16S, 18S and 23S as well as screen for pathogen-specific polymorphisms [11]. One of the challenges of this approach is the frequent and unexpected amplification of contaminating template DNA, as observed in control reactions. Another potential problem with targeting 16S rRNA pathogen specific sequences is unexpected polymorphisms. Hence, naturally occurring variants may not be represented on the microarray, and failure to detect the variants would represent false negatives [11]. Another common PCR based approach detects pathogen type by amplification of a specific set of genetic markers that are measured on an array that has several probes for genes from a set of organisms. Such tests have been used for food-borne bacteria such as *E. coli *O157:H7 [12], viruses [13] and mixtures of pathogens [14]. The drawback of using this approach with multiplex PCR primers sets is the generation of spurious products [11]. Array based technologies using 70-mer oligonucleotide probes derived from pathogen specific genes have similar factors that require consideration. For instance, viral detection using a microarray composed of 1,600 unique viral oligonucleotides (70-mers) derived from 140 distinct viral genomes has been previously demonstrated [15] as a powerful viral detection mechanism, but the drawback of this strategy is that only the group of known pathogen-specific genes will be queried.

Given the enormous spectrum of genetic possibilities, only a highly parallel field deployable technology that is universal in nature has near-term potential to address these needs. The initial vision for a universal DNA microarray was a matrix of oligonucleotide containing features with unique n-mer probes [16]. This matrix could in theory be used to query a biological sample for the presence of any nucleic acid sequence. This technique requires constructing an array that contains 4^n ^features. Larger values of n infuse greater specificity into the arrayed probes, but as n increases the number of required features grows rapidly. This universality is obtained by synthesizing a combinatorial n-mer array containing all 4^n ^possible sequences of length n [17]. The key issue is to find a value of n that is large enough to afford sufficient hybridization specificity, yet small enough to be practically fabricated and analyzed.

We have previously demonstrated the utility of a genome sequence-independent microarray for identifying genetic differences [18,19]. The initial prototype of universal arrays used oligonucleotide probe lengths of 12 and 13 bases. From 4^12 ^possible probes, a subset of 14,283 probes was synthesized using *in situ *synthesis technology and digital optical chemistry (DOC) [20-22]. Fluorescently labelled genomic DNA was hybridized to produce unique informative patterns (i.e. bio-signatures) on a test set of pathogens and host (*Bacillus subtilis, Yersinia pestis, Streptococcus peumoniae, Bacillus anthracis*, and *Homo sapiens*). In addition, we have shown that a custom microsatellite microarray can be used to demonstrate global differences between species by measuring hybridization intensities for every possible repetitive nucleotide motif from 1-mers to 6-mers [19]. Further we have used genome sequence independent microsatellites to identify global differences in the genomes of 93 cancer, cancer-free and high risk patient cell line samples [23]. This paper describes a larger high density oligonucleotide microarray with 370,000 elements, called Universal Bio-signature Detection Array (UBDA), designed by our laboratory and commercially produced by Roche-Nimblegen (Madison, WI) using light-directed photolithography [16,24]. The platform design which consists mainly of probes, that are tailored to be genome independent, is mathematically derived and therefore unbiased (Additional file 1, Table S1).

This strategy exploits the unique signature of a sample in the form of a pattern generated from hybridization of any unknown genome (DNA or cDNA) to a very high-density species-independent oligonucleotide microarray. *Brucella *species and several other pathogens were used as examples to demonstrate this forensics technology platform. Hybridization patterns are unique to a genome, and potentially to different isolates or a mixture of organisms. These techniques may be especially useful in evaluating and differentiating species whose genome has not yet been sequenced.

## Results

### UBDA array sensitivity and specificity of probe hybridization

**Figure 1:**
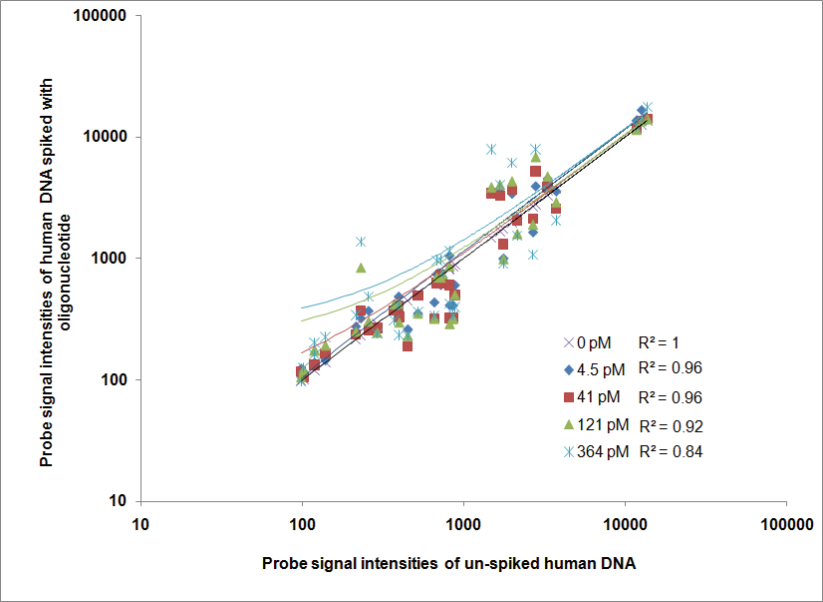
**Array sensitivity determined by control probe signal intensity values**. Human genomic DNA spiked with 70-mer oligonucleotides at different concentrations was compared against the same sample without oligonucleotides. Normalized signal intensity values from the Cy3 channel were plotted on a log scale and compared using linear regression from human genomic DNA samples with and without 70-mer oligonucleotides spiked into the labelling reaction. The probes being assessed on this scatter plot are perfect matches to the 70-mer oligonucleotide sequence. Each notation on the graph represents a specific concentration of spiked-in 70-mer oligonucleotides on an individual array. The oligonucleotides were spiked into the labelling reaction at a concentration range from 4.5 pM to 364 pM. The divergence of R^2 ^value from that with no spike-in was used to measure the sensitivity of detection on the array.

DNA microarrays using oligonucleotides are widely used in biological research and are usually sequence specific. Two primary types of parameters are required to evaluate the robustness and sensitivity of DNA microarray experiments- labelling and hybridization [16]. Sensitivity of a given array platform is often defined as the minimum signal detected by the array scanning system [25]. In our case we have used labelling controls, where specified DNA molecules (70-mer oligonucleotides) are spiked into experimental human genomic DNA samples prior to fluorescent labelling. A set of six synthetic 70-mer oligonucleotides (Additional file 2, Table S2) was designed to be spiked into each labelling reaction and hybridized to a constellation of 361 probes that were replicated five times on the array. We compared signal intensity values from control probes on the array hybridized with human genomic DNA and 70-mer oligonucleotides spiked into a separate sample of human genomic DNA. Each spike-in concentration was added on an individual array. We measured sensitivity of the array as a decrease in the correlation coefficient R^2 ^value in the signal intensity from human genomic DNA spiked with 70-mer oligonucleotides when compared to the unspiked human genomic DNA sample. The sensitivity of the UBDA was examined by the addition of 70-mer synthetic oligonucleotides to the labeling reaction of human genomic DNA sample (Cy-3 label). Spike-in control synthetic 70-mer oligonucleotides were added at varying concentrations; 4.5 picomolar, 41 picomolar, 121 picomolar and 364 picomolar respectively. Figure 1 elucidates that the sensitivity range of detection for the UBDA is between 364 picomolar and 121 picomolar as seen by the decreased R^2 ^values of 0.84 and 0.92 respectively for perfect match probes for these two concentrations when compared to the un-spiked human genomic DNA sample. The sensitivity of detection is estimated between a concentration of 364 picomolar and 121 picomolar. At concentrations lower than 121 picomolar, the R^2 ^value for perfect match probes is 0.96 which is within the ability to resolve samples statistically and confirms that there was no detectable variation at the lower oligonucleotide spike-in at these concentrations. This evaluation demonstrates the variability of signal intensities contributed by differences in oligonucleotide concentrations spiked into the human DNA sample compared to the un-spiked human DNA sample. Regression analysis of probe signal intensity values from the mis-matched probes in the data set are in Additional file 3, Figures S1A-S1D. We have assessed array variability over several arrays using a common human DNA sample in the reference channel. We obtained an R^2 ^value of 0.94 +-0.06.

The specificity of the computationally derived 9-mer probes on the UBDA array was studied using the selectivity of the middle nucleotide in each probe. We hypothesized that DNA strands generally will not hybridize efficiently to any probe for which there are multiple mismatches in proximity to the center-most base. The array design was based upon the prediction that the use of relatively short probes (15-21 mers) would result in the middle approximately 9 bases dominating hybridization kinetics. Probes on the UBDA that contained the StuI site (AGG^CCT) were located and classified by the nucleotide position of the cut point, relative to the center of the probe on the microarray by a custom computer code. DNA was digested to completion with StuI, and compared to matched DNA that was not digested. Each of the 9-mer probes with StuI restriction enzyme sites were binned depending on the nucleotide position of the StuI restriction site relative to the center of the probe. Thus probes with the StuI restriction enzyme site were binned in terms of base location according to the position of the StuI restriction enzyme cut site with respect to the center of the probe. As expected, probes with restriction enzyme site in the center of the probe displayed the highest degree of specificity demonstrated by a reduction in signal. A log_2 _fold change of -0.23 was obtained when comparing digested DNA to undigested DNA, averaged over microarray probes with the restriction enzyme site at the center of the probe. Microarray probes with the StuI site located at the center demonstrated reduced intensity, confirming specificity of genomic DNA to hybridize to the center of the probe. The trend of the log_2 _fold change increased as the StuI restriction enzyme site moved away from the center of the probe with the average results increasing towards zero (Additional file 4, Figure S2). Thus, confirming that the center nucleotide is the most selective in the hybridization complexes.

### Identification of synthetically mixed pathogen sample

**Figure 2:**
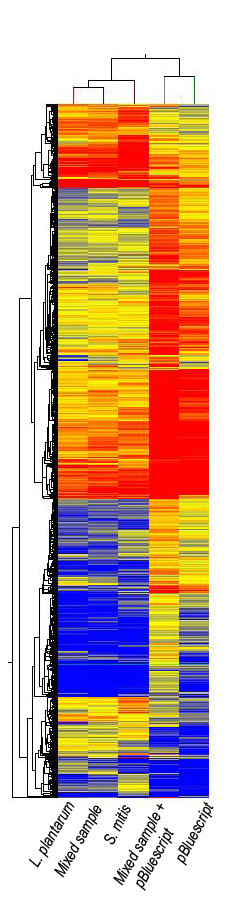
**Hierarchical clustering of mixed samples demonstrates the resolution capabilities of the UBDA array**. This dendogram and heat map illustrates a unique bio-signature pattern obtained from ***Lactobacillus plantarum***, mixed sample (synthetic mixture in a 4:1 ratio of ***L. plantarum and Streptococcus mitis***), ***S. mitis***, mixed sample (a synthetic mixture of ***L. plantarum ***and ***S. mitis ***genomic DNA in a ratio of 4:1 with a spike-in of pBluescript plasmid at 50 ng) and pBluescript plasmid. Normalized data from the 9-mer data set were filtered for intensity signals greater than the 20^th ^percentile. Only intensity signals with a fold change of 5 or greater were included. These 36,059 elements were subjected to hierarchical clustering with Euclidean distance being used as a similarity measure. The signal intensity values were represented on a log_2 _scale and range from 8.4 to 13.4.

To establish the ability to decipher a synthetically mixed sample on the UBDA array, *Lactobacillus plantarum *[GenBank accession number ACGZ00000000, genome size 3,198,761 bases] and *Streptococcus mitis *[26] [Genbank accession number FN568063, genome size 2,146,611 bases] genomic DNA were mixed in a ratio of 4:1 (2.53 × 10^8 ^copies of *L. plantarum *to 0.57 × 10^8 ^copies of *S. mitis *genomes) for a total of 1 μg of DNA, and thus adjusted for copy number of each of the two genomes and hybridized to the array. In addition, pure genomic DNA samples from *L. plantarum *and *S. mitis *were also hybridized individually on separate arrays. The minimum amount of sample required to be detected by hierarchical clustering was determined by an assumption that the mixed sample would cluster under the same node with known samples. As seen from Figure 2, the mixed sample comprising of *Lactobacillus plantarum *and *Streptococcus mitis *groups with pure samples from *L. Plantarum *and *S. mitis *(as shown in Figure 2, lane 1, 2 and 3). These results show that if 25% of the sample is from a second genome, it will group with the higher copy genome on the dendogram heat map generated from the hierarchical clustering algorithm. A sample with *Lactobacillus plantarum *and *Streptococcus mitis *genomic DNA in a 4:1 ratio (2.53 × 10^8 ^copies of *L. plantarum *to 0.57 × 10^8 ^copies of *S. mitis *genomes) was spiked-in with 50 ng (1.54 × 10^10 ^copies) of pBluescript plasmid (3,000 bases) [27]. However the node for this sample (Figure 2, lane 4) did not cluster with pure samples from *Lactobacillus plantarum *and *Streptococcus mitis*, instead it clustered closest to a pure sample of pBluescript (Figure 2, lane 5). Spike-in from a low complexity plasmid genome with a high copy number genome such as pBluescript can dominate the signature pattern. The alteration of the signature pattern is so great that the sample cannot be distinguished on the dendogram from pure bacterial genomes. Therefore, we are in the process of developing algorithms which will produce a similarity score for a given genome in a mixed genome sample by comparing it to a wide spectrum of species in our genome signature repository.

### Identification of genetic signatures from closely related *Brucella *species

The spectrum of organisms chosen for hybridization on this array, were primarily bio-threat zoonotic agents infecting farm animals. Our initial studies were based on the ability of the 9-mer probe signal intensities to distinguish between different *Brucella *species. Currently, there are nine recognized species of *Brucella *based on host preferences and phenotypic preferences. Six of those species are *Brucella abortus *(cattle), *Brucella canis *(dogs), *Brucella melitensis *(sheep and goat), *Brucella neotomae *(desert wood rats), *Brucella ovis *(sheep) and *Brucella suis *(pigs) [28]. All of these species are zoonotic except *B. neotomae *and *B. ovis*. Raw signal values from the pair data files for the Cy3 channel were background corrected and quantile normalized [29]. Signal intensities related to the 9-mer data set were parsed from the data file using a PERL script. These files were imported into the GeneSpring GX (Agilent, Santa Clara, CA) program. Data from these files was clustered using the hierarchical clustering algorithm to generate a heat map and identify a pattern within the underlying data.

**Figure 3:**
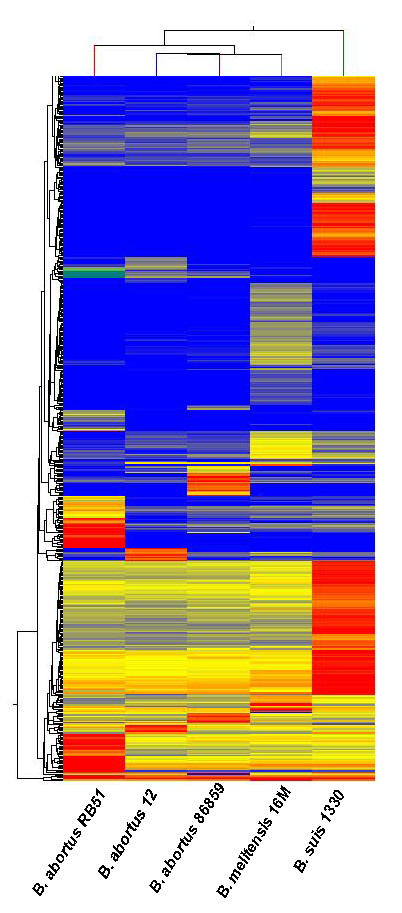
**Unique 9-mer probe bio-signatures from hybridization of *Brucella *genomes demonstrates ability to resolve highly similar genomes**. This dendogram illustrates the unique bio-signature obtained from ***Brucella abortus RB51***, ***Brucella abortus ***12, ***Brucella abortus ***86-8-59, Brucella ***melitensis ***16 M and Brucella ***suis ***1330. Normalized data from the 9-mer data set were filtered for intensity signals greater than the 20^th ^percentile. Only intensity signals with a fold change of 5 or greater were included. These 2,267 elements were subjected to hierarchical clustering with Euclidean distance being used as a similarity measure. The signal intensity values were represented as a log_2 _scale. The range of log_2 _values are from 7.2 to 13.

The dendogram of this heat map which runs vertically along the left side of the heat map in Figure 3 shows the unique bio-signature patterns from 9-mer probes obtained from *Brucella suis *1330, *Brucella abortus *RB51, *Brucella melitensis *16 M, *Brucella abortus *86-8-59 and *Brucella abortus *12. Normalized data from the 9-mer data set were filtered for intensity signals greater than the 20^th ^percentile. Only intensity signals with a fold change of 5 or greater were included. These 2,267 elements were subjected to a hierarchical clustering algorithm with Euclidean distance being used as a similarity measure. Centroid linkage rule was applied in the clustering algorithm. The signal intensity values were represented as a log_2 _scale. One of the array features was pathogen specific probes designed for independent validation. These probes are species specific to a small set of pathogens including Avian Influenza Virus, Rift Valley Fever Virus, Foot and Mouth Disease Virus, *Brucella melitensis *16 M, *Brucella suis *1330 and *Brucella abortus *biovar 1 strain 9-941 (Additional file 1, Table S1).

**Figure 4:**
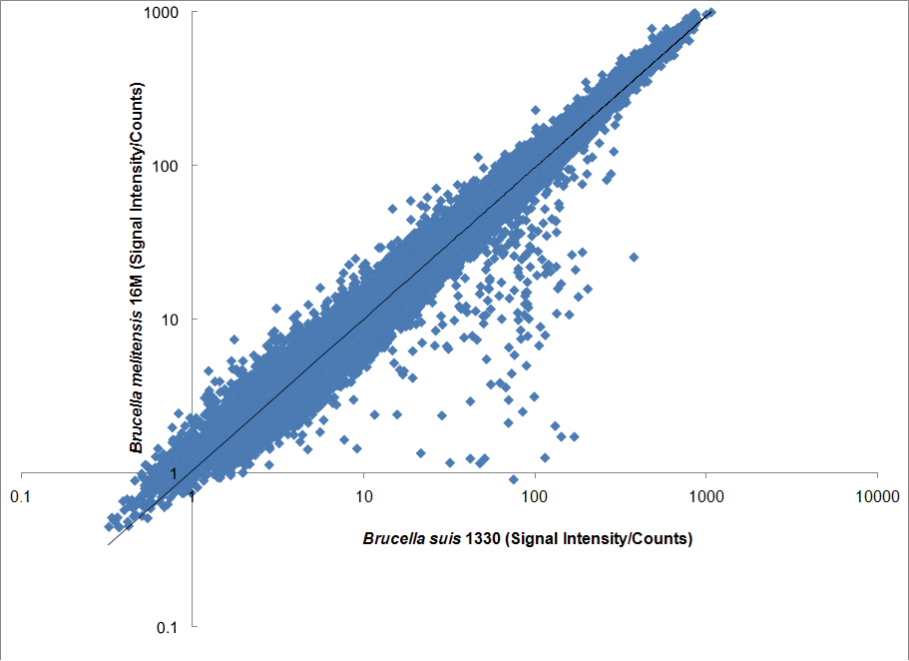
**Correlation of *Brucella Suis 1330 *and *Brucella melitensis 16 M *was computed by a ratio of signal intensity divided by counts of 9-mer probe occurrences in the respective genomes**. Normalized signal intensity for each of the 262,144 9-mer probes represented on the array were divided by the corresponding counts of 9-mer probe occurrences in the respective genome sequences for both ***B. suis ***and ***B. melitensis***. The resulting values for a set of 32,000 probes were then plotted, with ***B. melitensis ***and ***B. suis ***(signal intensity/counts) on the ordinate and abscissa, respectively. Pearson's correlation coefficient was subsequently calculated (ρ = 0.93 as shown).

The genomes of *B. melitensis *and *B. suis *have been completely sequenced (28, 29). Comparative genome analysis for these genomes shows that the two genomes are extremely similar. The sequence identity for most open reading frames (ORFs) was 99% or higher [30]. We computationally evaluated the published genome sequences for *B. suis *1330 [30] and *B. melitensis *16 M [31] to determine the specific instances in the genome sequence of each 9 base core probe sequence from the array. Normalized signal intensity for each of the 262,144 9-mer probes represented on the array were divided by the corresponding counts of 9-mer probe occurrences for both *B. suis *and *B. melitensis*. The resulting values for a set of 32,000 probes were then plotted as illustrated in Figure 4, with *B. melitensis *and *B. suis *(signal intensity/counts) on the ordinate and abscissa, respectively. Pearson's correlation coefficient was subsequently calculated (ρ = 0.93 as shown). This correlation value indicates that the 9-mer probe signal intensities are in agreement with 'known' genome sequence similarity scores for *B. melitensis *and *B. suis*.

### Taxonomic phylogenetic relationships between organisms hybridized on the UBDA array

**Figure 5:**
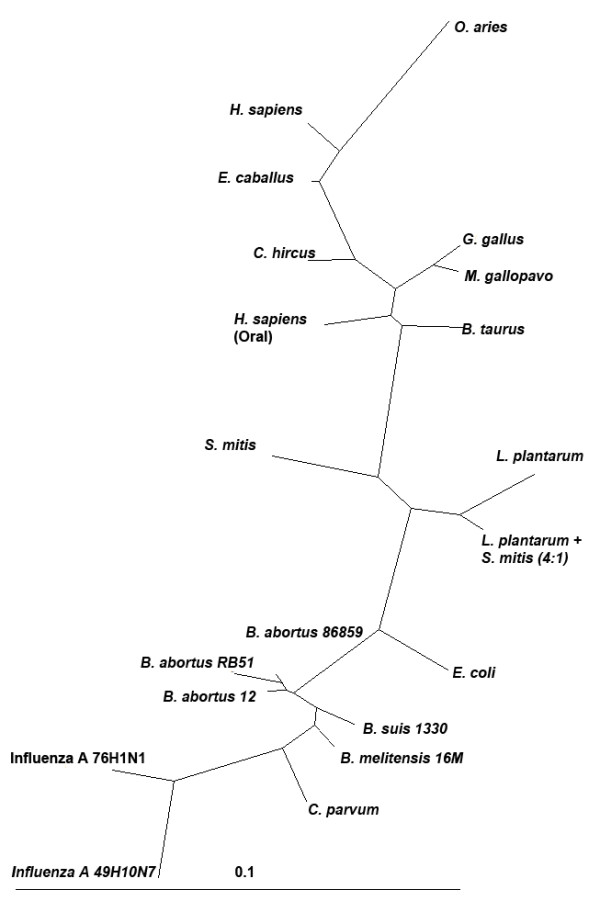
**Phylogenetic relationships from the 9-mer probe set between organisms hybridized on the UBDA array**. All 262,144 9-mer data points for each of the 20 samples were RMA normalized and log_2 _transformed. A Pearson correlation matrix was created by comparing each sample against all other samples. The values were used to generate a taxonomic relationship tree using the PHYLIP software. The taxonomic tree, as visualized in the Treeview program, shows the separation between mammalian, bacterial and viral genomes.

Phylogenetic trees are used as a tool in comparative sequence analysis to illustrate the evolutionary relationships among sequences. To create a phylogenetic tree based on 9-mer signal intensities, genomes listed in (Additional file 5, Table S3) were compared pair-wise, using the Pearson correlation measure (Figure 5). In this study, we demonstrate the use of signal intensities generated from 9-mer probe data to clearly cluster hosts and pathogens into to their 'known' phylogenetic relationships. We have previously shown that a custom microsatellite microarray can be used to demonstrate global microsatellite variation between species as measured by array hybridization signal intensities. This correlated with established taxonomic relationships [19]. Data obtained from the UBDA arrays (normalized signal intensity values) and computational analysis (log_2 _transformed, computed counts within sequenced genomes), for all 262,144 9-mer probes, were treated identically for the purposes of tree building. All 262,144 9-mer data points for each sample were first normalized using GeneSpring (percentile shift normalization followed by baseline to median normalization). A Pearson's correlation matrix was subsequently produced and then converted to a taxonomic tree using the neighbour-joining program within the PHYLIP software suite and TreeView program [32]. Trees were not rooted to any specific organism. The lower branches of the phylogenetic tree as shown in Figure 5 display the segregation and differentiation of the various *Brucella *species. The mixed sample comprising of *L. Plantarum *and *S. Mitis *(4:1 ratio) was found to be closer to the *L. Plantarum *(ρ = 0.974) versus *S. mitis *(ρ = 0.957) on the phylogenetic tree since there was a higher copy number of this genome in the sample (Figure 5). The tree illustrates that the 9-mer probe intensities can be used in species differentiation. The taxonomic tree is an approximate visualization estimation, using a distance matrix which successfully separated mammalian, bacterial and viral clades.

### Samples subjected to DNA amplification are comparable to unamplified samples

**Figure 6:**
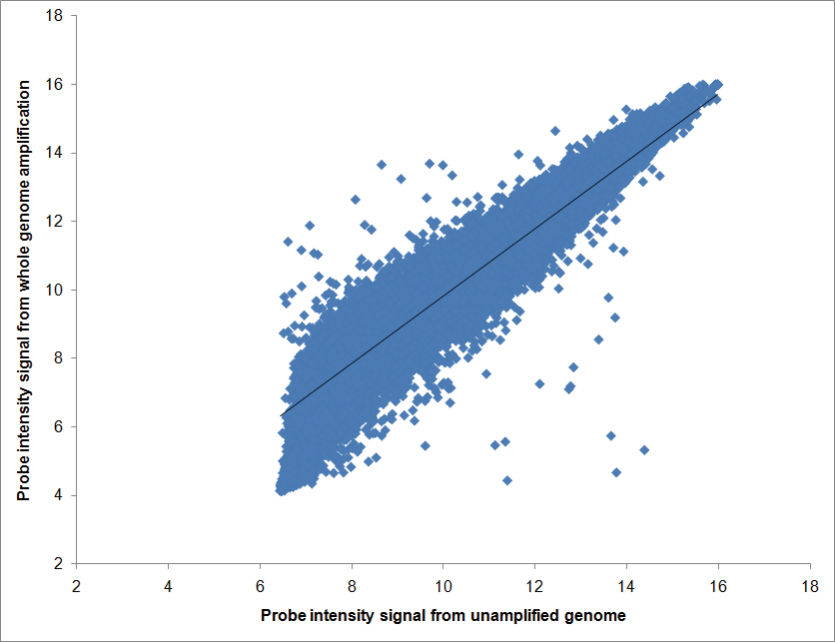
**Bivariate Fit of *Francisella tularensis *whole genome amplified genomic DNA (log_2 _values) by unamplified genomic DNA (log_2 _values)**. A linear regression of the two samples resulted in an R^2 ^value of 0.91, confirming that whole genome amplification of pathogen material such as ***Francisella tularensis ***LVS genomic DNA in small amounts (10 ng starting material) is comparable to the unamplified genomic sample.

In preparation for the UBDA becoming not only a detection assay but also a diagnostic test for the identification of numerous pathogens, it was recognized that pathogens may be present in a given sample at very low copy numbers and may be further diluted by genetic material recovered from the host. Microarrays require 0.5 - 1 μg of high-purity genomic DNA, which may be difficult to obtain from all samples. To overcome this limitation the potential for DNA amplification, artefacts that may significantly alter hybridization to the microarray were examined. To analyze for this possible limitation, a 10 ng (4.89 × 10^6 ^copies) aliquot of *Francisella tularensis *LVS strain genomic DNA [Accession number NC_007880, genome size 1,895,994 bases] was amplified using the whole genome amplification method (GenomiPhi V2, GE Healthcare). A total of 1 μg of the resulting amplified DNA was hybridized to the UBDA array and compared to the hybridization pattern resulting from the hybridization of 1 μg of unamplified DNA from the same source. Figure 6 shows a linear regression of the two samples (all 262,144 probes) which resulted in an R^2 ^value of 0.91, well within the R^2 ^= 0.94 +- 0.06 reproducibility found for the custom microsatellite microarray [19]. This confirms that whole genome amplification of pathogen material in small amounts is comparable to the unamplified genomic sample. We obtained these results using the standard protocol with 10 ng of starting material without optimization. We are targeting a 1-2 nanogram sample size as a starting amount of material in an optimized robust, field sample evaluation.

## Discussion

This is a new forensics array based technology to identify any species. This unique strategy of using patterns generated from hybridization of any unknown genome (DNA or cDNA) to a very high-density species independent oligonucleotide microarray and comparing those patterns to a library of patterns of known samples can be used to identify unknown organisms. Figure 5 shows the grouping of the different genomes into bacterial, viral and eukaryotic genomes. Further the *Brucella *species grouping pattern obtained from the phylogenomic analysis using the Pearson's correlation matrix shown in Figure 5 are in agreement with *Brucella *species showing hierarchical clustering represented as a similarity matrix shown in Figure 3. The UBDA hybridization patterns are unique to a genome, and potentially to different isolates and to a mixture of organisms. In the future, this forensics method will work by comparing signal intensity readout to a library of readouts established by interrogating a wide spectrum of species which will be available at our website http://discovery.vbi.vt.edu/ubda/. The phylogenetic tree illustrates the ability of 9-mer probes to differentiate among *Brucella *species. Pair-wise comparisons between different genomes can be used as a measure to classify bacterial, viral or mammalian genomes into their respective clades. We have begun to amass a library of 'signatures' to facilitate accurate identification and classification of "unknown" samples. We are currently expanding the repository of available bio-signatures to several hundred genomes including field isolates from bacteria, viruses, host genomes and vectors infected with pathogens. Some of the genomes in this repository are classified in the select agent category. UBDA forensics application has the potential to be compatible with micro-machine based front end sample processing and preparation platforms, thus enabling the production of a highly automated, fast and accurate field-deployable detection system.

Other diagnostic techniques such as PCR or RT-PCR require several primers to be designed which are specific for each genome- bacterial, viral or host. There may be spurious products for primers binding at low specificity. The processing costs should also be taken into consideration for these methodologies. The current cost for the UBDA array is approximately $350 per sample which includes reagents and processing costs. The current turnaround time for this forensics technology is less than 24 hours. This is a single experimental procedure compared to other technologies which involve a series of methods such as serological, biochemical and genomic based. Genome specific arrays are in the similar price range as the UBDA array; however researchers can only assay a single genome or a small subset of them. Currently the UBDA platform requires a turnaround time approximately one day from hybridization on the array to data analysis. A diagnostic laboratory in the field requires proximately two weeks before the identity of a given infectious agent can be determined. These methods usually require several standard serological and biochemical tests that are usually selected and based on the clinical symptoms observed in the field. Serology test results are usually available after 48 hours. Although each of these tests is cost effective in nature, they must be fine tuned to be pathogen specific.

The UBDA approach can be applied to any genome, even in the presence of background contamination (usually host DNA) for which, the unique pattern will be known. The patterns generated from an unknown sample (secretion, tissue culture, environmental sample, etc) with minimal specimen processing can be identified or at least the most similar related species will be predicted by comparison to a library or a repository of patterns. These techniques may be especially useful in evaluating and differentiating species whose genome has not yet been sequenced. Along with a repository of unique hybridization signatures from the genomes of pathogens and their hosts, this array has the ability to rapidly and adequately identify biological threat agents and newly emerging infectious pathogens that are high risk priorities in bio-defense. Application of this technology has the potential to extend to other areas such as food and environmental microbial monitoring and basic research including, (a) speciation and evolution, (b) human/animal disease biomarker discovery, (c) measurement of the genomic response to a chemical, radiation or other exposure, but most important, (d) pathogen forensics and characterization of natural or engineered variants that may confound other species-specific approaches.

## Conclusions

Genetic signature discovery and identification of pathogenic phenotypes will provide a robust means of discriminating pathogens that are closely related. This array has high sensitivity as demonstrated by the detection of low amounts of spike-in oligonucleotides. Hybridization patterns are unique to a specific genome and these can be used to de-convolute and thus identity the constituents of a mixed pathogen sample. In addition it can distinguish hosts and pathogens by their divergent phylogenomic relationships as captured in their respective 9-mer hybridization signatures. This platform has potential for commercial and government agency applications as a cost effective reliable platform for accurately screening large numbers of samples for bio-threat agents in forensic analysis, screening for pathogens that routinely infect animals and humans, and as a molecular diagnostic of micro-organisms in a clinical environment. This platform is highly attractive, because it has multiplex capacity where knowledge can be drawn from the array hybridization patterns without prior explicit information of the genomes in the samples. These hybridization patterns are being translated into a knowledge base repository of bio-signatures so that future users of this technology can compare and draw inferences related to the sample under study. The data from these experiments and the array design are located on our web site at http://discovery.vbi.vt.edu/ubda/.

## Methods

### Array design details

A custom microarray was designed by this laboratory and manufactured by Roche-Nimblegen (Madison, WI) as a custom 385 K (385,000 probe platform) chip to include the following sets of probes; 9-mer, pathogen specific probes; rRNA gene specific, microsatellite and control 70-mer oligonucleotide probes. There were 262,144 9-mer probes, and 20,000 of them were replicated 3 times in total (Additional file 1, Table S1). The 9-mer probes were comprised of a core 9-mer nucleotide and flanked on both sides by three nucleotides, selected to maximize sequence coverage of these basic 15-mers. Probes with low GC content were padded with additional bases at their termini to equalize melting temperatures, with most probes ranging from 15-21 nucleotides in total length. For the 9-mer design, the length of the probes was adjusted to match a melting temperature of 54°C. The array design was based upon the prediction that the use of relatively short probes (15-21 mers) would result in the middle 9 bases dominating hybridization kinetics.

rRNA probes were included in the design to serve as positive controls and confirmation of the 9-mer probes power for differentiating genomes. The rRNA probes were selected from the green gene data (http://greengenes.lbl.gov/cgi-bin/nph-show_probes_2_otu_alignments.cgi), utilizing the complete list of 8,935 OTUs (Operational Taxonomic Unit). One probe was selected for each OTU and probe length was adjusted to a T_m _equal to 54°C, as was done for 9-mer design. A mis-match probe (1 mis-match, MM) for each OTU probe was included in the design. Perfect match (PM) 8,935 probes and 8,935 one mis-match MM probes were included in the microarray design. All probes are replicated 3 times on the array.

Genome specific probes for *Brucella *spp., Avian Influenza Virus (AIV), Foot and Mouth Disease Virus (FMDV), and Rift-Valley Fever Virus (RVFV) were designed and included on the microarray as an independent test when the array is used to analyze these species. Sequence alignments were performed to determine the similar and unique regions of the pathogens, with probes selected from the unique regions of each pathogen species or sub-type, and excluding sequences similar to host genomes. In total, 1,062 unique probes were selected and are replicated 3 times.

Probes dedicated to surveying microsatellite content were designed for every 1- to 6-mer repetitive sequence. For each 1- to 5-mer repetitive sequence, single mis-match (1 MM) probes were also designed. A total of 3,557 unique microsatellite probes were generated and replicated at total of 3 times. Microsatellite probes were included on this array to anchor the results to previous experiments and to aid in the de-convolution of the contribution of host genomic DNA. For higher life forms typically have many microsatellite loci, whereas bacteria and viruses have none or very few in their genome.

Gene-specific probes were designed to target important metabolic pathways, such as alcohol dehydrogenase, glucose-6-phosphate isomerase and SHV-like β-lactamase, by using the highly conserved sequences. In total, 432 probes were designed and replicated a total of 3 times.

For labelling controls, a set of six synthetic 70-mer oligonucleotides were designed to be spiked into each labelling reaction and hybridized to a constellation of 361 dedicated probes on the array comprising of perfect match probes (34 probes), 1 mis-match (100 probes), 2 mis-match (137 probes) and 3 mis-match probes (90 probes), ranging from 15-19 nucleotides. The set of 361 probes are replicated 5 times total (Additional file 2, Table S2). Figure 1 shows a comparison of signal intensity values of perfect match control probes on the array generated from human genomic DNA without spike of oligonucleotides to samples with a spiked-in. Regression analysis of signal intensity values from the mis-matched probes on the data set is in Figures S1A-S1D (Additional file 3). The array design files for each feature category on the UBDA array are in Additional file 6 (9-mer probes) and Additional file 7 (all other probes) and also available at http://discovery.vbi.vt.edu/ubda/.

### Microarray procedure

Human genomic DNA was extracted from blood samples collected from a volunteer by the McDermott Center for Human Growth and Development Genetics Clinical Laboratory in accordance with Institutional Review Board at UT Southwestern Medical Center (Dallas, TX). Genomic DNA from *Bos taurus*, *Gallus gallus*, *Meleagris gallopavo*, *Ovis aries*, *Capra hircus*, and *Equus caballus *was obtained from Zyagen (San Diego, CA). *Brucella *species, *Cryptosporidium parvum, Lactobacillus plantarum*, *Streptococcus mitis*, *Escherichia coli *and Influenza virus genomic DNA was obtained from BEI resources and ATCC (Manasses, VA). The spectrum of organisms chosen for hybridization on the UBDA array was primarily bio-threat zoonotic agents, agents infecting farm animals.

DNA concentration (260 nm) and purity (260/280 and 260/230 nm) was assessed by the spectrophotometer and quality by agarose gel electrophoresis. Samples with 260/230 nm ratios greater than 1.8 were used following established protocols for array comparative genomic hybridization (CGH). Hybridization conditions were optimized to ensure specificity and sensitivity. All DNA test samples (1 μg) were labelled with Cy3 and co-hybridized with the same Cy5-labeled human reference (Promega, Inc, Madison, WI), according to Roche Nimblegen standard microarray labelling procedures. For each microarray, human genomic DNA (Promega, Madison, WI) was labelled with Cy-5 and used as a reference channel in each experiment. DNA labelling, hybridization and data acquisition were performed by Mogene (St. Louis, MO). We tested hybridization temperatures ranging from 30°C to 50°C. For microarray hybridization, a custom buffer (0.5% Triton X-100, 1 M NaCl, and 100 mM Tris-HCl pH 7.5, filtered with a 0.2 micron nitrocellulose filter, prepared fresh) was used at 38°C, and microarrays were washed following Roche Nimblegen's CGH standard techniques (available at http://www.nimblegen.com). Hybridization conditions were standardized for the UBDA array to minimize any errors that could lead to bias resulting after processing the slides and image scanning on an array scanner. Signals from probes complementary to labelling controls indicate that the post-DNA preparation process, from labelling to hybridization, washing and scanning, were successful. Hybridization, scanning, and data extraction were performed following Roche NimbleGen standard protocol for CGH arrays, and the resulting raw data were provided via secure web link.

### Array data processing and organism classification

A Robust Multi-chip Average (RMA) normalization procedure was performed across all arrays. The procedure included background subtraction and quantile normalization using Nimblescan Software (Roche NimbleGen). After normalization, all 262,144 9-mer probes were extracted from the 370 K array using PERL scripts and averaged across the replicate probes. Subsequent statistical analysis was performed using GeneSpringGX 11.0 (Agilent Technologies, Santa Clara, CA). All signal intensity values were log_2 _transformed for further analysis. Data were also filtered by intensity values (lower cut off percentile of 20% for raw signals), and subsequent pair-wise comparisons were performed on the sample data set. Clustering is one of the data mining processes for discovery and identifying patterns in the underlying data. Clustering algorithms partition data into subsets based on similarity and dissimilarity. Clustering methods follow three steps: pattern recognition, use of a clustering algorithm and similarity measure matrix [33]. For pattern recognition, pair-wise comparisons are used between samples to select the features on which the clustering is to be performed. Our experimental platform is comparative genome hybridization for which hierarchical clustering is used to determine phylogenomic relationships between organisms. Hierarchical clustering [34] transforms a distance matrix of pair-wise similarity measurements between all items into a hierarchy of nested groupings. The hierarchy is represented with a binary tree-like dendogram. Hierarchical clustering was performed on the resulting data sets, using the Euclidian matrix and centroid linkage to classify various organisms. Data sets were analyzed for *Brucella *species. A cut-off of 5-fold change in hybridization intensity for a given probe was used to reduce the data set to only those meaningful probes that showed a difference between at least one of the pair-wise comparisons.

### Phylogenetic taxonomic tree based on array intensity

Data obtained from the Universal Bio-Detection Array (normalized signal intensity values that were log_2 _transformed) and computational analysis for all 262,144 9-mer probes were treated identically for the purpose of tree building. All 262,144 data points for each of the 20 samples were first RMA normalized. For each sample, a Pearson's correlation matrix was created which included self similarity and similarity to the remaining 19 samples from all the 262,144 data points of each sample. The resulting distance matrix was used to produce a phylogenetic tree, using the neighbour-joining method within the PHYLIP software suite and TreeView.

### Whole genome amplification

*Francisella tularensis *LVS strain genomic DNA, starting material, 10 nanogram was amplified using whole genome amplification method as defined (GenomiPhi V2, GE Healthcare). We obtained 2-3 μg of whole genome amplified DNA from 10 ng of starting genomic DNA.

## Authors' contributions

SJS oversaw the project, coordinated the study design, carried out the analysis and subsequent parsing and data interpretation and drafted the manuscript. JNW initiated the project, participated in preliminary technical analyses. CLG participated in manuscript editing. LM participated in manuscript editing, created the UBDA website and provided computation expertise. ZS designed the array and provided computation expertise. JM provided useful discussions and technical assistance. LGA provided DNA samples, data interpretation and participated in manuscript editing. HRG conceived of the study, participated in the study design and mentored in drafting the manuscript. All authors have agreed to all the content in the manuscript, including the data as presented.

## Supplementary Material

Additional file 1 **Table S1 Distribution of probe types included in the UBDA design**. The table describes the different data set features on the array. Click here for file

Additional file 2 **Table S2 Sequence of labelling control oligonucleotide probes**. Sequence information of the 70-mer oligonucleotides used in the spike-in study to determine the sensitivity of the UBDA array. Click here for file

Additional file 3 **Figures S1A - S1D**. Regression analysis of signal intensity values generated from spike in of different concentrations of 70-mer oligonucleotides to human genomic DNA versus the un-spiked sample. Average Cy3 signal intensity values were plotted on a log scale. Normalized signal intensities from the Cy3 channel, which were human genomic DNA samples with and without the addition of 6 spike-in 70-mer oligonucleotides, were compared by linear regression. Each notation on the graph represents an individual control probe spot on the array. The R^2 ^value is displayed in the lower right quadrant of the graph. Purple × represent perfect match probes (PM), blue diamonds represent 1 mis-match (MM) probes, red squares represent probes with 2 mis-matches and green triangles represent 3 mis-matches. (A) At 4.5 picomolar of oligonucleotide spike-in, an R^2 ^value of 0.96 was obtained for probes with a PM, 0.93 for 1 MM, 0.95 for 2 MM and 0.92 for 3 MM. (B) At 41 picomolar of oligonucleotide spike-in, an R^2 ^value of 0.96 was obtained for probes with a PM, 0.87 for 1 MM, 0.94 for 2 MM and 0.86 for 3 MM. (C) At 121 picomolar of oligonucleotide spike-in, an R^2 ^value of 0.92 was obtained for probes with a PM (perfect match), 0.85 for 1 MM, 0.90 for 2 MM and 0.83 for 3 MM. (D) At 364 picomolar of oligonucleotide spike-in, an R^2 ^value of 0.84 was obtained for probes with a PM (perfect match), 0.81 for 1 MM, 0.90 for 2 MM and 0.75 for 3 MM. Blast searches were done for all 70 mer probe combinations to the human genome sequence. The 2 MM 70-mer oligonucleotide probes were highly similar to the human genome and hence are not considered informative and do not show any variation as represented by the linear regression value. Click here for file

Additional file 4 **Figure S2**. Analysis of probe hybridization specificity on the UBDA array. Human genomic DNA was digested with StuI (AGG^CCT) restriction enzyme, and then compared to undigested human genomic DNA from the same individual. The resulting values were plotted, with ratio of the human genomic DNA digested with StuI and undigested human genomic DNA as log_2 _fold change on the ordinate axis. The nucleotide position of the StuI restriction enzyme site relative to the center of the 9-mer probe is plotted on the abscissa axis. Probe specificity analysis of individual 9-mer probes is confirmed by demonstrating that the center most base governs the hybridization kinetics. This is shown by a reduction in probe signal intensity values when the human genomic DNA sample was digested with StuI enzyme. The reduction in the probe intensity signal is greater when the restriction enzyme site is located at the center of the 9-mer probe. Therefore the center nucleotide of the probe is the most restrictive in determining the specificity of the probe hybridization complex. Click here for file

Additional file 5 **Table S3 Genomes hybridized on the array**. Genomic DNA from the following genomes was hybridized on the UBDA array. Click here for file

Additional file 6 **Annotation file for 9-mer probes on the UBDA array**. Click here for file

Additional file 7 **Annotation file for all other probes on the UBDA array**. Genomic DNA from the following genomes was hybridized on the UBDA array. Click here for file
