# Supplementary material for: A species independent universal bio-detection microarray for pathogen forensics and phylogenetic classification of unknown microorganisms
Source: BMC Microbiol. 2011 Jun 14;11:132. doi: 10.1186/1471-2180-11-132 (PMC3130645; doi:10.1186/1471-2180-11-132)
Supplement: Additional file 1 — Table S1 Distribution of probe types included in the UBDA design. The table describes the different data set features on the array. [file 1471-2180-11-132-S1.PDF]

Feature Category	Feature count with replicates	Description
**9-mer (4^9 probes equivalent to 262, 144 probes)**	302,144	Sequence independent including every 9 bp combination (20,000 probes replicated 3x total)
**rRNA**	53,634	Probes designed from 16s rRNA sequences (replicated 3x total)
**Gene specific probes**	1,296	Probes derived from alcohol dehydrogenase, glucose-6-phosphate isomerase and SHV-like β-lactamase (replicated 3x total)
**Pathogen specific probes**	3,186	Specific to *Brucella* spp., Avian Influenza virus, Rift Valley Fever Virus and Foot and Mouth Disease Virus (replicated 3x total)
**Microsatellites probes**	10,671	Every 1-mer to 6-mer repetitive sequences (replicated 3x total)
**70-mer oligonucleotide control probes**	1,805	Measure and calibrate labelling and hybridization efficiency and specificity (replicated 5x total)
**Randomer specificity probes for E. coli**	375	
**Total probes**	**373,111**	
