# Supplementary material for: A species independent universal bio-detection microarray for pathogen forensics and phylogenetic classification of unknown microorganisms
Source: BMC Microbiol. 2011 Jun 14;11:132. doi: 10.1186/1471-2180-11-132 (PMC3130645; doi:10.1186/1471-2180-11-132)
Supplement: Additional file 2 — Table S2 Sequence of labelling control oligonucleotide probes. Sequence information of the 70-mer oligonucleotides used in the spike-in study to determine the sensitivity of the UBDA array. [file 1471-2180-11-132-S2.PDF]

**Probe**	**Sequence**
1	CTACCTCCGATCGCGATACAGAATGAATCATGGGATTCATATTGAGACAGTT GTTCTGTCTTGGCTGGAC
2	ACCGACTAAAGGTAATGACCATTGGTGAATTGATACCGTCTACAACCCTCCA ATGTTACAAGAGACTAAC
3	AATGGAAAAGTTGGCTCCGGGTCTTACACCTGCGTGCCTCGATGCTAACAG ACCCCAGGGCGACCGATAT
4	TGTCAGACCGTAGCGTTGCAGCTTCAGTCACACAGCTTTGGCTTAGAGATTC CGCCAAAAGAACCATCCT
5	ATGCGTATGCTGCAACCAACGATTAATCCGGTCTCCTATAGGACATCGCGAT AAGATCGTCTAACGTAGC
6	GGACCGCTAGTTGTCGGACCATACATTGATGTTGGAATATGCGGATACCCA GGCAATCATTTCACCTTTT
