# Supplementary figures and images for: A species independent universal bio-detection microarray for pathogen forensics and phylogenetic classification of unknown microorganisms
Source: BMC Microbiol. 2011 Jun 14;11:132. doi: 10.1186/1471-2180-11-132 (PMC3130645; doi:10.1186/1471-2180-11-132)

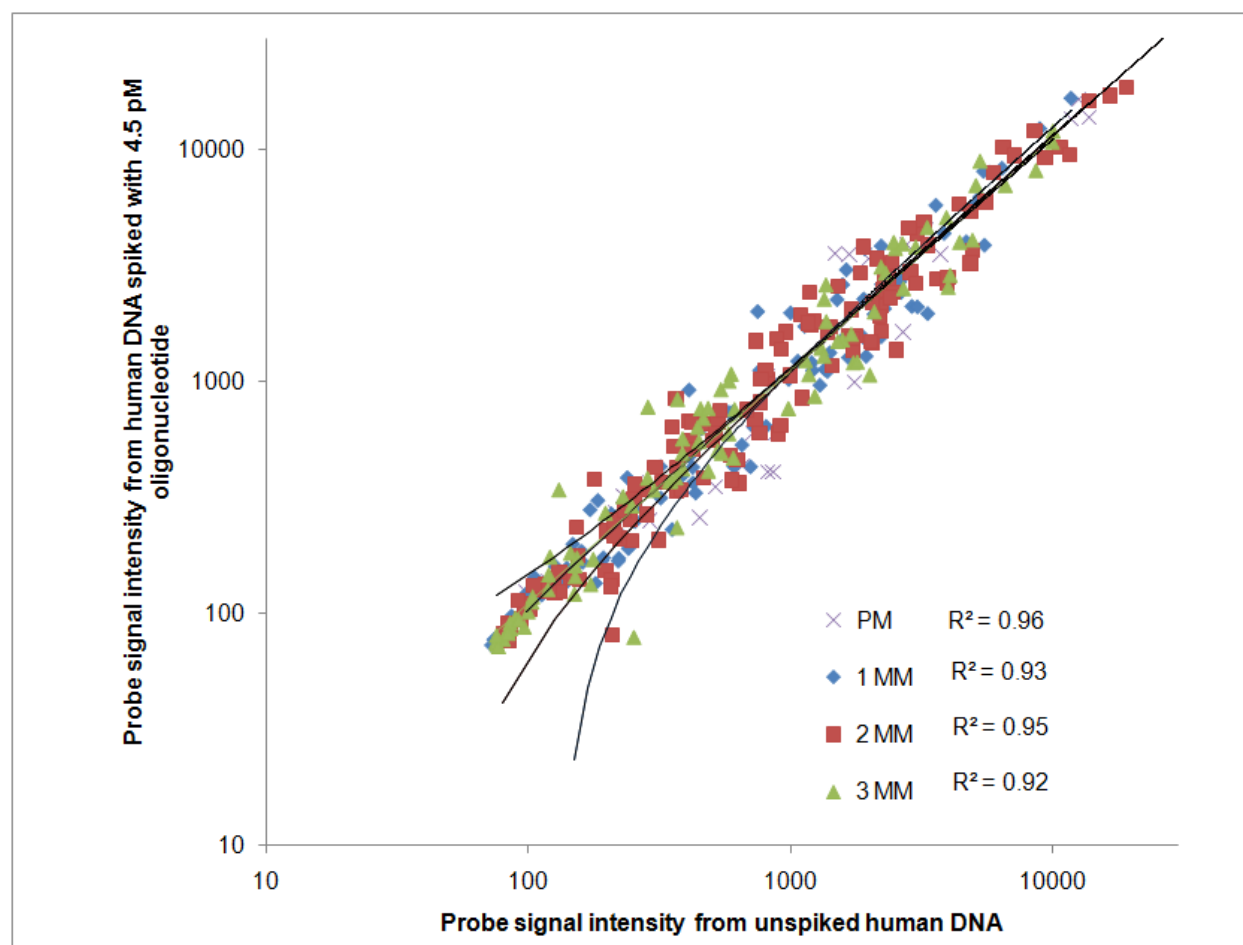

**Figure S1A**

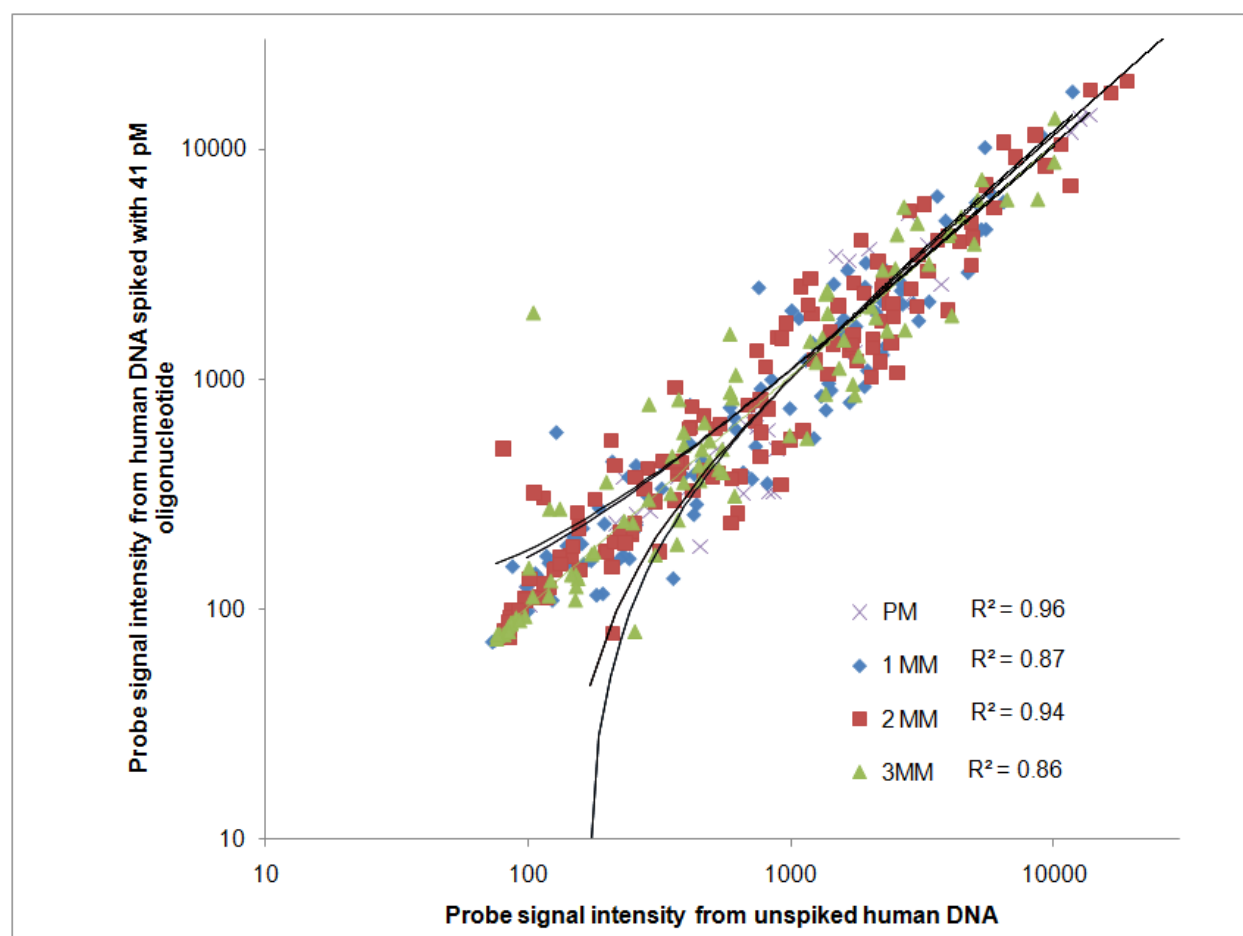

**Figure S1B**

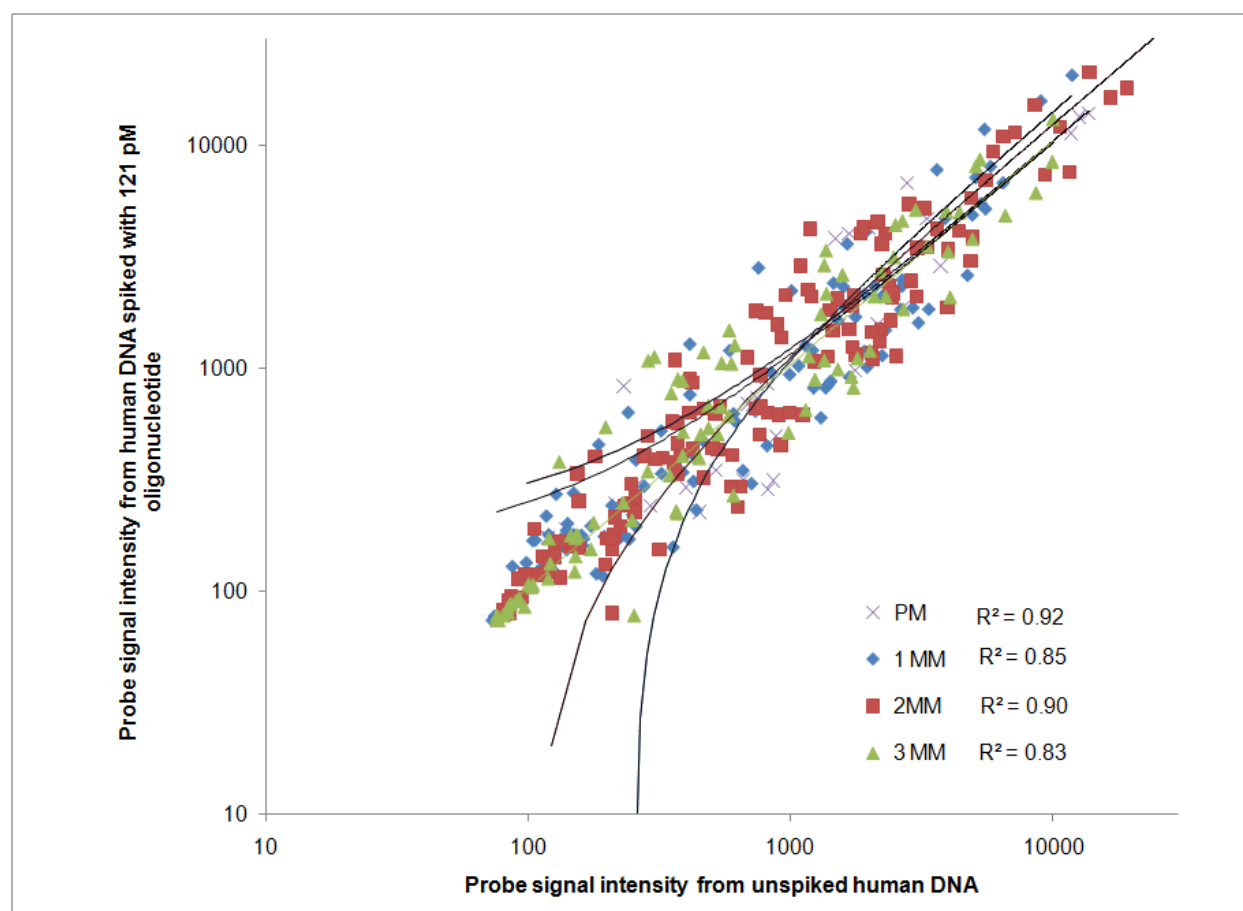

**Figure S1C**

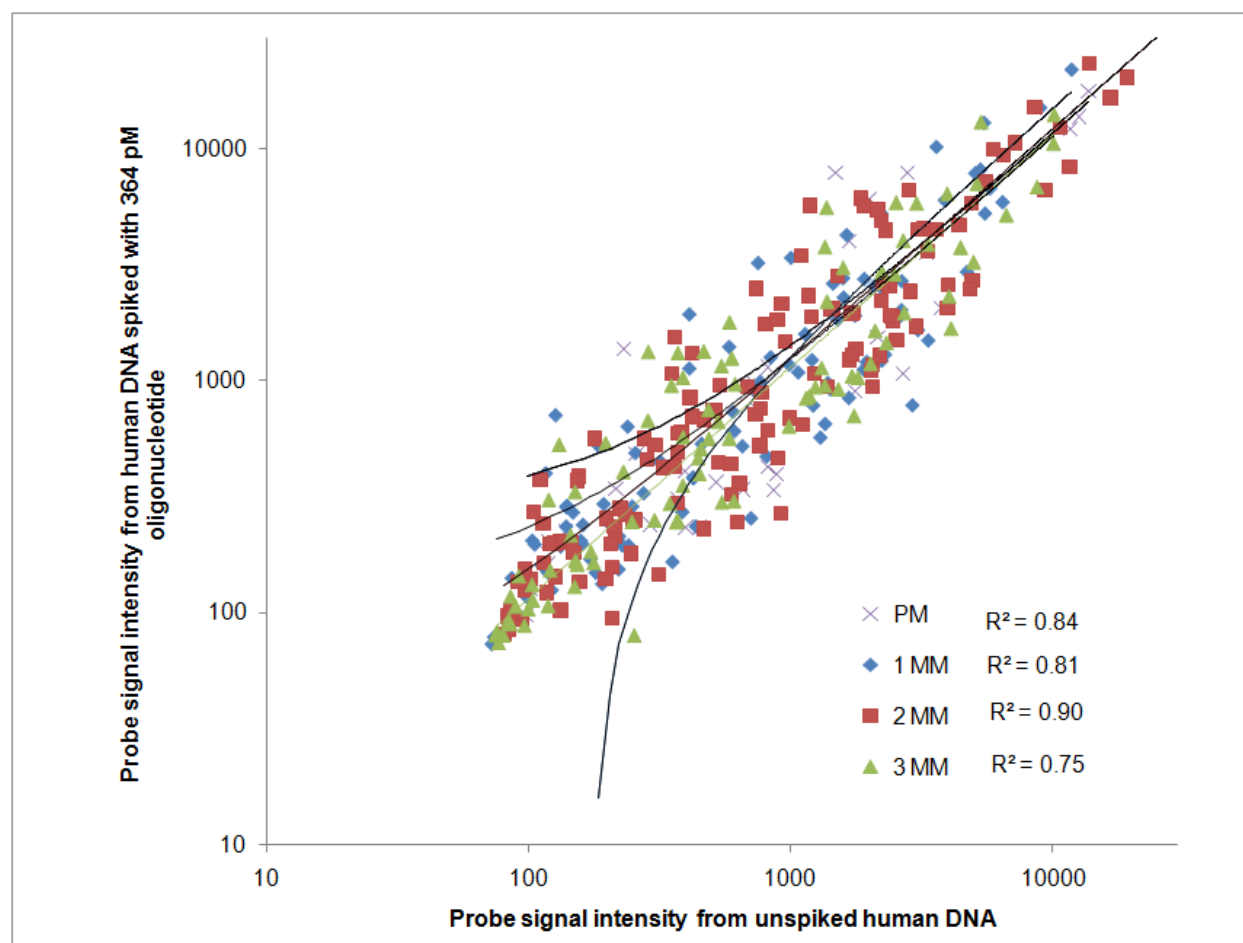

**Figure S1D**

Supplement: Additional file 3 — Figures S1A - S1D. Regression analysis of signal intensity values generated from spike in of different concentrations of 70-mer oligonucleotides to human genomic DNA versus the un-spiked sample. Average Cy3 signal intensity values were plotted on a log scale. Normalized signal intensities from the Cy3 channel, which were human genomic DNA samples with and without the addition of 6 spike-in 70-mer oligonucleotides, were compared by linear regression. Each notation on the graph represents an individual control probe spot on the array. The R2 value is displayed in the lower right quadrant of the graph. Purple × represent perfect match probes (PM), blue diamonds represent 1 mis-match (MM) probes, red squares represent probes with 2 mis-matches and green triangles represent 3 mis-matches. (A) At 4.5 picomolar of oligonucleotide spike-in, an R2 value of 0.96 was obtained for probes with a PM, 0.93 for 1 MM, 0.95 for 2 MM and 0.92 for 3 MM. (B) At 41 picomolar of oligonucleotide spike-in, an R2 value of 0.96 was obtained for probes with a PM, 0.87 for 1 MM, 0.94 for 2 MM and 0.86 for 3 MM. (C) At 121 picomolar of oligonucleotide spike-in, an R2 value of 0.92 was obtained for probes with a PM (perfect match), 0.85 for 1 MM, 0.90 for 2 MM and 0.83 for 3 MM. (D) At 364 picomolar of oligonucleotide spike-in, an R2 value of 0.84 was obtained for probes with a PM (perfect match), 0.81 for 1 MM, 0.90 for 2 MM and 0.75 for 3 MM. Blast searches were done for all 70 mer probe combinations to the human genome sequence. The 2 MM 70-mer oligonucleotide probes were highly similar to the human genome and hence are not considered informative and do not show any variation as represented by the linear regression value. [file 1471-2180-11-132-S3.PDF]

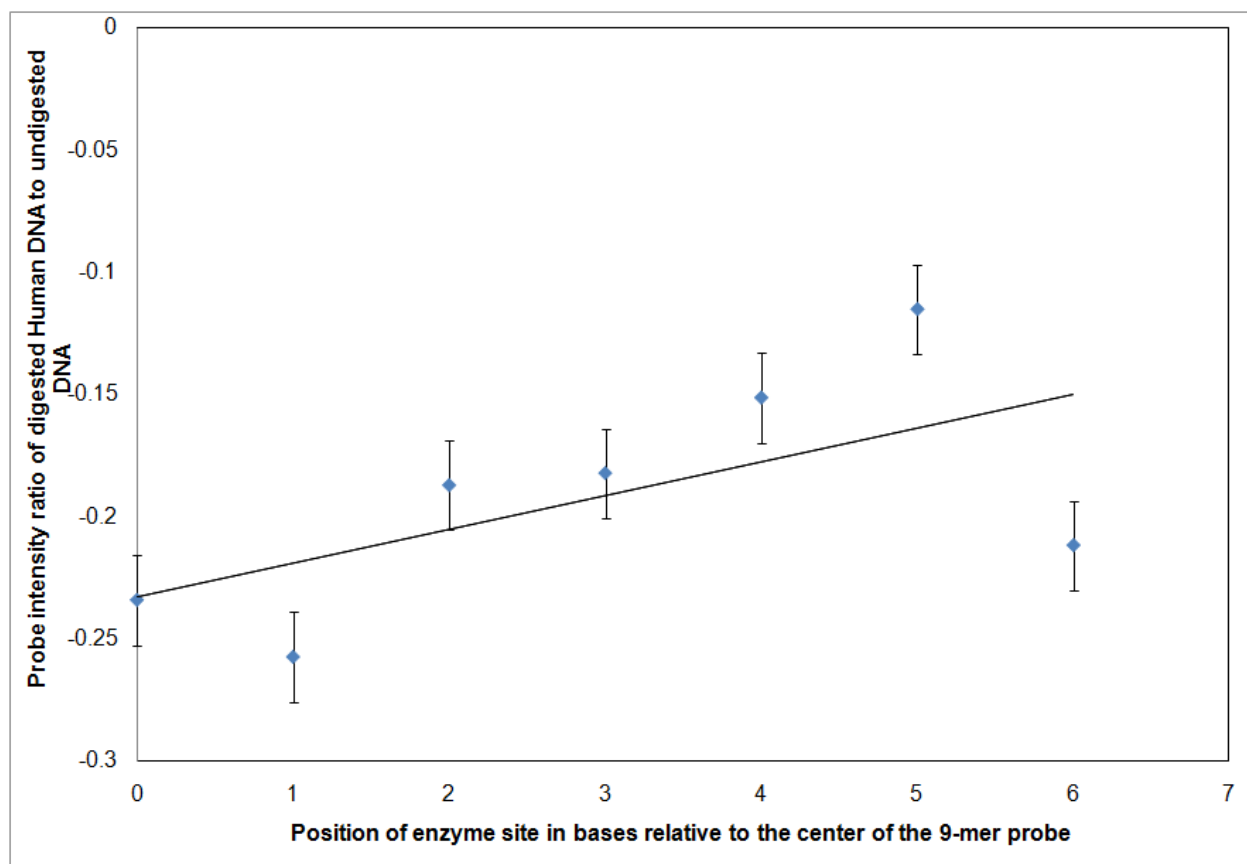

Supplement: Additional file 4 — Figure S2. Analysis of probe hybridization specificity on the UBDA array. Human genomic DNA was digested with StuI (AGG^CCT) restriction enzyme, and then compared to undigested human genomic DNA from the same individual. The resulting values were plotted, with ratio of the human genomic DNA digested with StuI and undigested human genomic DNA as log2 fold change on the ordinate axis. The nucleotide position of the StuI restriction enzyme site relative to the center of the 9-mer probe is plotted on the abscissa axis. Probe specificity analysis of individual 9-mer probes is confirmed by demonstrating that the center most base governs the hybridization kinetics. This is shown by a reduction in probe signal intensity values when the human genomic DNA sample was digested with StuI enzyme. The reduction in the probe intensity signal is greater when the restriction enzyme site is located at the center of the 9-mer probe. Therefore the center nucleotide of the probe is the most restrictive in determining the specificity of the probe hybridization complex. [file 1471-2180-11-132-S4.PDF]
