# Supplementary material for: A species independent universal bio-detection microarray for pathogen forensics and phylogenetic classification of unknown microorganisms
Source: BMC Microbiol. 2011 Jun 14;11:132. doi: 10.1186/1471-2180-11-132 (PMC3130645; doi:10.1186/1471-2180-11-132)
Supplement: Additional file 5 — Table S3 Genomes hybridized on the array. Genomic DNA from the following genomes was hybridized on the UBDA array. [file 1471-2180-11-132-S5.PDF]

**Eukaryotes**	**Prokaryotes**	**Viruses**
Homo sapiens (Human)	*Lactobacillus plantarum*	*Influenza A 49H10N7*
Bos taurus (Bull)	*Escherichia coli* K12	*Influenza A 76H1N1*
Gallus gallus (Chicken)	*Brucella abortus* RB51	
Meleagris gallopavo (Turkey)	*Brucella abortus* 12	
Ovis aries (Sheep)	*Brucella abortus* 86859	
Capra hircus (Goat)	*Brucella suis* 1330	
Equus caballus (Horse)	*Brucella melitensis* 16M	
Cryptosporidium parvum		
